# Challenges of Green Transition in Polymer Production: Applications in Zero Energy Innovations and Hydrogen Storage

**DOI:** 10.3390/polym16101310

**Published:** 2024-05-07

**Authors:** Iva Rezić, Ernest Meštrović

**Affiliations:** 1 Department of Applied Chemistry, Faculty of Textile Technology, University of Zagreb, 10000 Zagreb, Croatia; 2 Faculty of Chemical Engineering and Technology, University of Zagreb, 10000 Zagreb, Croatia; emestrov@fkit.hr

**Keywords:** polymer, zero energy, innovation, hydrogen storage, green sustainable transition

## Abstract

The green transition in the sustainable production and processing of polymers poses multifaceted challenges that demand integral comprehensive solutions. Specific problems of presences of toxic trace elements are often missed and this prevents shifting towards eco-friendly alternatives. Therefore, substantial research and the development of novel approaches is needed to discover and implement innovative, sustainable production materials and methods. This paper is focused on the most vital problems of the green transition from the aspect of establishing universally accepted criteria for the characterization and classification of eco-friendly polymers, which is essential to ensuring transparency and trust among consumers. Additionally, the recycling infrastructure needs substantial improvement to manage the end-of-life stage of polymer products effectively. Moreover, the lack of standardized regulations and certifications for sustainable polymers adds to the complexity of this problem. In this paper we propose solutions from the aspect of standardization protocols for the characterization of polymers foreseen as materials that should be used in Zero Energy Innovations in Hydrogen Storage. The role model standards originate from eco-labeling procedures for materials that come into direct or prolonged contact with human skin, and that are monitored by different methods and testing procedures. In conclusion, the challenges of transitioning to green practices in polymer production and processing demands a concerted effort from experts in the field which need to emphasize the problems of the analysis of toxic ultra trace and trace impurities in samples that will be used in hydrogen storage, as trace impurities may cause terrific obstacles due to their decreasing the safety of materials. Overcoming these obstacles requires the development and application of current state-of-the-art methodologies for monitoring the quality of polymers during their recycling, processing, and using, as well as the development of other technological innovations, financial initiatives, and a collective commitment to fostering a sustainable and environmentally responsible future for the polymer industry and innovations in the field of zero energy applications.

## 1. Introduction

The presence of trace impurities in polymers poses several challenges in the context of a sustainable economy. Firstly, there is an environmental impact. It is known that trace impurities in polymers can have adverse environmental consequences when released into the environment during manufacturing, use, or disposal [1,2,3]. These impurities may leach out of the polymer matrix and contaminate the soil, water, and air, leading to pollution and ecosystem damage.

Secondly, health concerns are significant. Many impurities in polymers may pose risks to human health. For example, certain dyes and catalysts used in polymer production may contain toxic substances that can migrate into the food or beverages stored in plastic containers, potentially causing health problems upon consumption.

Thirdly, there are also significant recycling challenges since trace impurities can complicate the recycling process of polymers. Contaminants present in recycled plastics can degrade material quality, reduce mechanical properties, and limit the range of applications for which the recycled material can be used. This hinders the effectiveness of recycling efforts and undermines the circular economy’s principles.

Moreover, due to more and more strict regulatory compliance standards, it is quite possible that the materials we do not consider to be problematic to the environment and human health come up on the unwanted lists of chemicals. The presence of trace impurities in polymers may raise even more concerns regarding regulatory compliance with safety and environmental standards. Manufacturers may face regulatory scrutiny and legal obligations to ensure that their products meet stringent quality and safety requirements, which can entail additional costs and administrative burdens.

Last, but not least, there is a consumer perception that strongly influences marketing possibilities. If noted, trace impurities, in terms of the toxic organic or inorganic compounds evidenced in polymers, can erode consumer confidence in the sustainability and safety of products. The public awareness of the environmental and health issues associated with plastic pollution is increasing, leading consumers to demand more transparent and eco-friendly products. Any perception of risk or uncertainty regarding trace impurities can deter consumers from purchasing polymer-based products [4,5,6].

**Figure 1:**
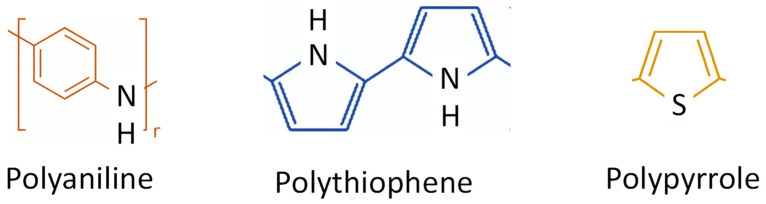
Conducting polymers for hydrogen storage: polyaniline, polypyrrole, and polythiophene.

Such polymers should enable safe hydrogen storage, and conducting polymers (shown in Figure 1) have recently emerged as promising materials on the horizon that are suitable for this purpose due to their significant advantages: their low cost, simple steps in synthesis and processing (the preparation and shaping due to aromatic rings resulting in (i) high resonance energy and stability, making them less reactive, the (ii) tendency to undergo substitution reactions rather than addition reactions, and (iii) the delocalized pi-electrons which are spread out over the entire ring, contribute to stability), the achievable different tailoring in their morphology and architecture, and the possibility of doping and composite formation that enable their enhanced performance through modification. Moreover, such materials are chemically stable and offer interesting functional properties. For all of those reasons, those materials are suitable for hydrogen storage and fuel cell membranes.

Addressing these challenges requires concerted efforts from stakeholders across the polymer value chain. Strategies to mitigate the impact of trace impurities include adopting cleaner production processes, implementing stringent quality control measures, developing safer alternatives to hazardous additives, improving recycling technologies, and enhancing public awareness and education on sustainable consumption practices. By addressing trace impurities in polymers, we can move towards a more sustainable economy that prioritizes environmental protection, human health, and resource conservation [7,8,9].

**Figure 2:**
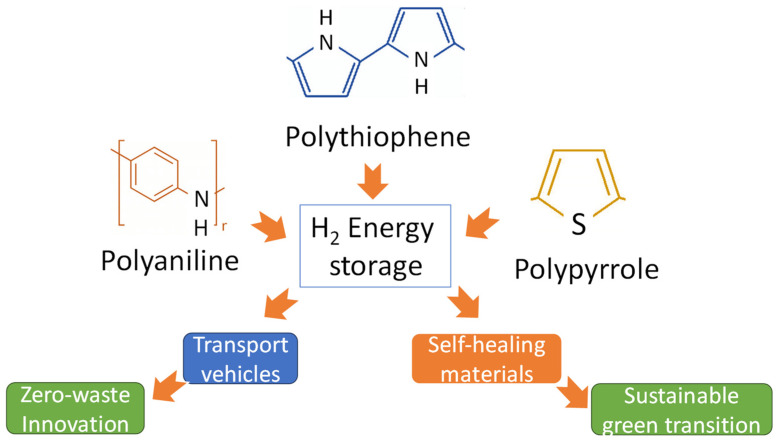
Conducting polymers in zero-waste innovations and green, sustainable transitions.

In addition, the application of polymers for zero-energy innovations in hydrogen storage and green, sustainable transitions presents several challenges. Firstly, the development of polymers with high hydrogen storage capacities while maintaining their stability and durability is a significant and urgent topic. These polymers need to efficiently adsorb and release hydrogen under practical conditions, which requires precise control over their molecular structures and porosity [10,11]. Only after this can their properties be applied in zero-waste innovations and green, sustainable transitions (as is shown in Figure 2).

Additionally, the integration of these polymers into practical hydrogen storage systems poses engineering challenges, including the design of suitable containment materials and interfaces to prevent hydrogen leakage. Furthermore, ensuring the scalability and cost-effectiveness of polymer-based hydrogen storage technologies remains a key challenge, as the large-scale production of specialized polymers may require advanced manufacturing processes and materials [12,13,14,15]. Moreover, the compatibility of polymer-based storage systems with existing infrastructure and regulatory frameworks needs to be addressed to facilitate their widespread adoption in the transition towards a green and sustainable energy landscape.

Novel materials are needed in hydrogen storage. In the transition towards clean energy, this is a pathway to reducing greenhouse gas emissions and enhancing energy security across sectors like transportation and power generation. Its zero-emission profile, particularly when utilized in fuel cells, underscores its potential as an environmentally friendly energy carrier. The current development stage in this field includes several pathways of development: (i) methods such as compressed gas and adsorption on porous materials like metal–organic frameworks (MOFs), (ii) liquid hydrogen storage options, (iii) solid hydrogen storage options (through metal hydrides and carbon-based materials), and, particularly, (iv) polymers such as polyaniline, polypyrrole, polythiophene, hyper-crosslinked polymers, polymers of intrinsic microporosity, conjugated microporous polymers, porous aromatic frameworks, and carboxymethyl cellulose.

Overall, addressing these challenges will be essential for realizing the full potential of polymers in enabling zero-energy innovations for hydrogen storage and green, sustainable transitions.

## 2. Zero-Waste Energy: Towards a Sustainable Future

We have witnessed an era marked by environmental degradation and climate change, so the concept of zero-waste energy emerges as a beacon of hope for a sustainable future. Zero-waste energy entails the efficient utilization of resources, minimizing waste generation, and maximizing energy recovery from discarded materials. This chapter delves into the principles, technologies, challenges, and opportunities associated with zero-waste energy, highlighting its crucial role in mitigating environmental impacts and advancing towards a circular economy.

The principles of Zero-Waste Energy are simple, as at its core, zero-waste energy embodies the principles of waste prevention, reuse, recycling, and energy recovery. Waste prevention involves minimizing waste generation through conscious consumption and production practices. Reuse entails prolonging the lifespan of products and materials through repair, refurbishment, and repurposing. Recycling involves the conversion of waste materials into new products or raw materials, thereby closing the loop of resource utilization. Energy recovery focuses on harnessing energy from waste materials through processes such as combustion, anaerobic digestion, and thermal or biological conversion.

There is a wide variety of technologies used for Zero-Waste Energy, encompassing energy from various waste streams, ranging from municipal, solid waste to industrial, agricultural, and biomass residues. Anaerobic digestion processes convert organic waste into biogas, a renewable energy source rich in methane. Waste-to-energy (WtE) facilities utilize thermal treatment methods such as incineration or gasification to combust waste and generate heat or electricity. Advanced technologies such as pyrolysis and hydrothermal carbonization offer innovative approaches to convert organic waste into biochar or synthetic fuels. Additionally, emerging technologies like microbial fuel cells and enzymatic digestion hold promise for decentralizing energy production from organic waste [16,17,18,19].

Despite its potential benefits, the widespread adoption of zero-waste energy faces several challenges. Its economic viability, technological barriers, regulatory constraints, and public perceptions are among the key hurdles to overcome. Its economic feasibility remains a critical factor, as the cost of waste management and energy recovery must be balanced against potential revenue streams. Technological barriers include the need for improved efficiency, scalability, and compatibility with diverse waste streams. Regulatory frameworks must be conducive to incentivizing zero-waste practices and supporting the development of renewable energy infrastructure. Furthermore, public awareness and engagement are essential to garnering support for zero-waste initiatives and fostering a culture of resource conservation.

However, among these challenges is a huge field of opportunities for innovation, collaboration, and systemic change. Advances in technology, coupled with favorable policy incentives and public–private partnerships, can drive the transition towards a zero-waste energy future. Integrated waste management systems that prioritize source reduction, recycling, and energy recovery offer a holistic approach to waste valorization [20,21]. Furthermore, decentralized and community-based initiatives empower local stakeholders to participate in the transition towards sustainable waste management practices.

## 3. Sustainable Polymers for Hydrogen Storage and a Circular Polymer Economy

The concept of a circular polymer economy represents a paradigm shift in the management of plastic materials, aiming to minimize waste generation, maximize resource efficiency, and promote sustainable practices throughout the lifecycle of polymers. In contrast to the traditional linear economy model of “take-make-dispose”, a circular polymer economy prioritizes strategies such as reusing, recycling, and redesigning to create a closed-loop system where polymers are continually reused, remanufactured, or regenerated. This chapter explores the principles, challenges, opportunities, and implications of transitioning towards a circular polymer economy, highlighting its potential to mitigate plastic pollution, conserve resources, and foster a more sustainable future.

The principles of the circular polymer economy are the ideas of waste prevention, resource recovery, and circular design. Waste prevention involves minimizing the generation of plastic waste through measures such as product redesigning, material substitution, and extended producer responsibility. Resource recovery encompasses strategies such as recycling, mechanical and chemical recycling, and energy recovery to extract value from post-consumer and post-industrial plastic waste streams. Circular design emphasizes the importance of designing products and packaging with circularity in mind, considering factors such as material selection, recyclability, and end-of-life options to facilitate the ease of reuse, repair, or recycling.

**Figure 3:**
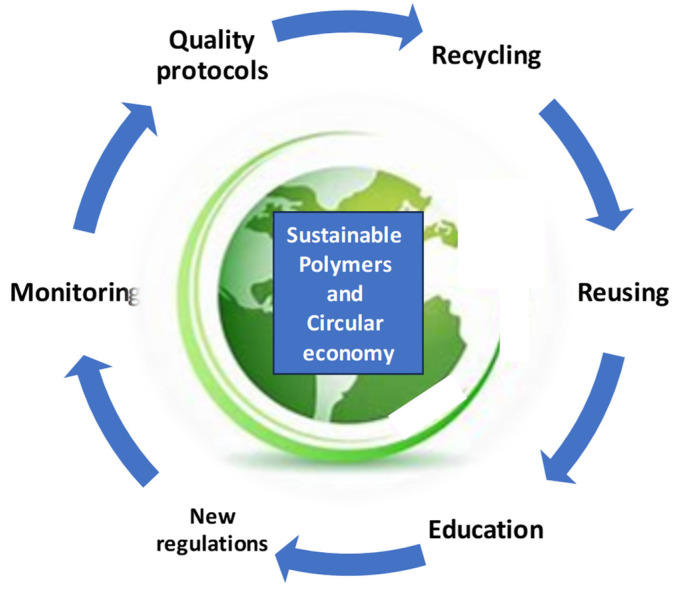
Sustainable polymers and a circular economy.

Transitioning towards a circular polymer economy is not without its challenges. A limited infrastructure, technological barriers, economic constraints, and consumer behaviors pose significant hurdles to overcome. Achieving high-quality recycling and closing material loops requires investment in recycling infrastructure, innovation in recycling technologies, and collaboration across the value chain. Furthermore, shifting consumer preferences towards sustainable products, incentivizing circular business models, and overcoming regulatory barriers are essential for driving systemic change. However, amidst these challenges lie opportunities for innovation, collaboration, and systemic transformation. Advances in material science, recycling technologies, and digitalization offer new avenues for enhancing resource efficiency and circularity in polymer production and consumption. However, without proper standard quality protocols, monitoring, education, and new regulations, it is hard to believe that proper reusing and recycling will be possible (Figure 3).

The implications of a circular polymer economy extend beyond its environmental benefits to encompass economic, social, and geopolitical considerations. By reducing the dependency on virgin fossil resources, promoting local recycling and manufacturing, and creating new market opportunities for recycled materials, a circular polymer economy can stimulate economic growth, job creation, and innovation. Moreover, fostering collaboration between stakeholders, empowering local communities, and promoting consumer awareness and engagement are crucial for realizing the full potential of circularity in polymer management.

The transition towards a circular polymer economy represents a fundamental shift in how we produce, consume, and manage plastic materials. By embracing the principles of waste prevention, resource recovery, and circular design, societies can mitigate plastic pollution, conserve natural resources, and create a more sustainable and resilient future. However, achieving a circular polymer economy requires concerted efforts from policymakers, industry stakeholders, and civil society to overcome challenges, capitalize on opportunities, and drive systemic change towards a more circular and sustainable polymer ecosystem.

Hydrogen has emerged as a promising alternative energy carrier due to its high energy density and potential for clean combustion [22,23,24,25,26,27,28]. However, the effective storage of hydrogen remains a key challenge for its widespread adoption in various applications, including transportation and renewable energy systems. Sustainable polymers offer a promising solution for hydrogen storage, leveraging their tunable properties, lightweight nature, and compatibility with existing infrastructures. The recent advancements, challenges, and opportunities in utilizing sustainable polymers for hydrogen storage, with a focus on their enhancing storage capacity, stability, and recyclability will be mentioned here [29,30,31,32,33,34,35,36,37].

There are many advantages in using polymers for hydrogen storage as sustainable materials [38,39,40]. The term “Sustainable” polymers encompasses a diverse range of materials derived from renewable resources or designed with eco-friendly synthesis routes. These polymers exhibit tunable properties such as their porosity, surface area, and chemical functionality, which can be tailored for efficient hydrogen adsorption and desorption. Porous polymers, including covalent organic frameworks, metal–organic frameworks as well as porous organic polymers, have garnered significant attention for their high surface area and controllable pore structures, enabling enhanced hydrogen storage capacities. Additionally, functionalized polymers incorporating metal or metal oxide nanoparticles demonstrate synergistic effects, facilitating hydrogen uptake through physisorption or chemisorption mechanisms [41,42].

**Figure 4:**
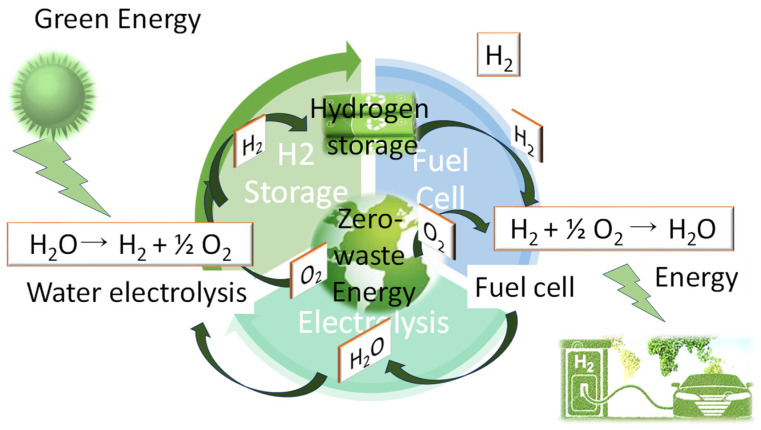
Sustainable zero-waste energy.

However, despite the promising advancements, several challenges hinder the practical implementation of sustainable polymers for hydrogen storage. Limited hydrogen uptake capacities, the slow kinetics of hydrogen adsorption/desorption, and the stability under operating conditions are among the key challenges to address [43,44,45,46,47,48]. Furthermore, the cost-effectiveness, scalability, and recyclability of sustainable polymer-based storage systems require optimization to compete with conventional storage technologies. However, ongoing research efforts focused on material design, structural optimization, and process engineering offer opportunities to overcome these challenges and unlock the full potential of sustainable polymers for hydrogen storage (Figure 4).

Recent trends have shown that future research directions in sustainable polymers for hydrogen storage encompass interdisciplinary approaches integrating materials science, chemistry, and engineering [49,50,51,52,53]. Tailoring polymer structures at the molecular level, exploring novel synthesis methodologies, and optimizing storage conditions hold promise for achieving higher hydrogen storage capacities and improved performance metrics [54,55,56,57,58,59,60,61,62,63,64,65,66,67,68,69,70,71,72,73,74,75,76,77,78,79,80,81,82,83,84,85,86,87,88]. Furthermore, the integration of sustainable polymer-based storage systems into hydrogen infrastructures, including onboard storage for fuel cell vehicles, stationary storage for renewable energy integration, and portable applications, presents exciting opportunities for real-world impact [89,90,91,92].

## 4. Detection of Trace Impurities in Polymers for a Sustainable Economy

As was previously emphasized, there are significant health concerns related to the trace amounts of impurities in polymer materials since many of those compounds pose risks to human health [93]. Dyes and catalysts used in polymer production may contain toxic substances that can migrate into the environment after disposal, or even into our food or beverages stored in plastic containers during the usage of polymers, potentially causing health problems upon the consumption of such foods or the exposure to such waste (in the land, air, and rivers) [94].

**Figure 5:**
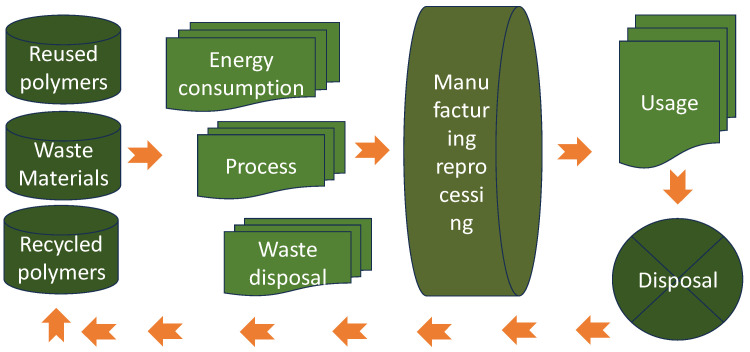
LCA of polymers.

The life cycle assessment describes the fate of the polymer materials in a sustainable economy (Figure 5).

The spectroscopic determination of impurities in polymers before recycling involves the use of various spectroscopic techniques to identify and quantify the contaminants present in polymer waste streams [95,96,97,98,99]. These impurities can include residual monomers, additives, pigments, fillers, and other substances that may affect the quality and performance of recycled polymers. Several spectroscopic methods are commonly employed for this purpose.

Infrared spectroscopy with Fourier transformation can be widely used to identify polymers’ functional groups and particular bonds of interest. For example, after the functionalization of the polymer surface via this method, it can be easily concluded if novel chemical bonds occurred or not. In addition, this methodology is used to detect impurities based on their characteristic absorption bands in the infrared spectrum, providing qualitative and semi-quantitative information about the composition of polymer samples.

The UV–Visible spectroscopy of polymers can be utilized to detect colored impurities, such as dyes, stabilization agents, and pigments. By measuring the absorption of light at specific wavelengths, UV–Vis spectroscopy can quantify the concentration of impurities and assess the color quality of polymer samples.

Fluorescence spectroscopy can be employed to detect fluorescent impurities in polymers. Certain contaminants may exhibit fluorescence when excited by light of a specific wavelength, allowing for their identification and quantification.

X-ray photoelectron spectroscopy is a technique that provides information about the chemical composition of polymer surfaces, usually used to identify surface contaminants and assess the cleanliness of polymer samples before recycling.

Raman spectroscopy offers molecular fingerprinting capabilities for the identification of impurities in polymers. It can detect changes in molecular structures and compositions, providing valuable information about the presence of contaminants in polymer samples.

By employing these spectroscopic techniques, recyclers can effectively analyze polymer waste streams for impurities and assess their impact on the quality of recycled materials. This information can inform process optimization, quality control measures, and material selection strategies to enhance the efficiency and sustainability of polymer recycling operations.

The analysis of toxic trace elements in polymers holds significant importance, not only for the purposes of reuse and recycling, but also for safeguarding the global population against the hazards of improper waste management. Experimental studies conducted on animals have demonstrated that several heavy metals, such as the elements presented in Table 1, can induce the development of cancers. Furthermore, epidemiological investigations have revealed a correlation between carcinogenesis in humans and exposure to such compounds, as outlined in Table 2.

The detrimental effects of heavy metals on human health encompass organ damage, respiratory organ and tract disorders, lung problems, cardiac problems, blood abnormalities, neurological disorders, skin ailments, as well as complications in fertility and pregnancy. The heavy metals present in human or animal bodies accumulate in different body tissues and, after this, they interact with enzymes which can disrupt cellular functions, potentially leading to the development of tumors or cancers. In response to these risks, various ecological textile standards have been implemented. These standards aim to ensure the safety of textiles and minimize the presence of harmful substances.

Some of the prominent ecological standards provide recommended limits for toxic elements, which are crucial for ensuring the safety and sustainability of textiles. These limits are outlined in Table 3 to guide manufacturers and consumers in making informed choices regarding textile products.

As can be seen from Table 2, the metals that accumulate in almost all systems are cobalt and lead, making them very problematic elements when present in polymer samples.

However, the toxic trace metals are not the only problems present in polymers, as there are other groups of problematic molecules, as is presented in Table 4.

The OEKO-TEX Standard is an initiative developed to provide consumers with safety and confidence in textile products by setting strict requirements for the ecology and human use of textiles.

This certification covers several product classes, including raw polymer materials, intermediate products, and finished products. Certification is based on extensive testing to ensure that polymer products are free from harmful chemicals and meet specific environmental standards.

## 5. Importance of Standardization Protocols and the Determination of Trace Impurities in Polymers in Regulatory Compliance and Risk Assessment

The accurate determination of heavy metal concentrations, particularly in trace amounts, is vital due to their potential hazards to human health and the environment. To achieve reliable and consistent results, the development of and adherence to standardized methods is crucial. The International Organization for Standardization (ISO) plays a pivotal role in establishing these standards, ensuring uniformity, comparability, and quality across laboratories worldwide. ISO standards provide guidelines and protocols for various analytical techniques used in detecting and quantifying heavy metals. These standards cover aspects such as sampling procedures, sample preparation, instrumental analysis, quality control measures, and data reporting. Standardization ensures that analytical methods yield accurate and precise measurements of heavy metal concentrations. These include the use of certified reference materials (CRMs) for calibration, regular performance checks, proficiency testing, and adherence to stringent data validation criteria.

The determination of elemental composition in polymer materials, in compliance with the standardized protocols, involves the application of various spectroscopic methods [100,101,102,103,104,105,106,107,108]. The selection of a particular spectroscopic method is influenced by several factors. These factors include the quantity of the sample and its characteristics. Regulatory agencies rely on the standardized methods endorsed by the ISO to assess their compliance with permissible limits of heavy metals polymers (Table 5). Furthermore, standardized analytical procedures facilitate risk assessment studies, allowing policymakers to make informed decisions regarding environmental protection and public health.

However, there is a lack of standards, as is emphasized in ISO/TR 23891:2020(en).

Plastics—their recycling and recovery—have a necessity of standards. Those which are relevant to the field (such as ISO/TC 61, Plastics, SC 14, Environmental aspects; CEN/TC 249, Plastics, ISO/TC 122/SC 3, Performance requirements and tests for means of packaging, packages and unit loads, ISO/TC 122/SC 4, Packaging and environment and CEN/TC 261/SC 4, Packaging and the environment, ASTM Subcommittee D20.95 on Recycled Plastics (USA), UNI (Italy), BIS (India), JISC (Japan); ISO/TR 22293:2021 Evaluation of methods for assessing the release of nanomaterials from commercial, nanomaterial-containing polymer composites; or ISO 15270:2008 Plastics—Guidelines for the recovery and recycling of plastic waste; or ISO/TR 21960:2020 Plastics—Environmental aspects—State of knowledge and methodologies) do not provide all necessary information to end users. For this reason, this paper goes beyond the current state-of-the-art standards in the field.

Despite efforts to develop safer alternatives, the dispersion of hazardous metals in modern consumer goods persists, particularly through material recycling practices.

The widespread presence of plastics in everyday life exacerbates the issue, leading to poor management and disposal practices that contribute to environmental contamination. Additionally, restricted additives have been observed in marine and industrial applications, further exacerbating the contamination of plastics in natural environments.

Unfortunately, there has been limited attention given to the presence of hazardous metals in environmental plastics. While there is a misconception that low concentrations of the metals acquired from the surroundings pose minimal risks, studies have shown that the concentrations of additive-bound metals in historical plastics can be significantly higher. Under conditions simulating the digestive environment, these metals can be mobilized from plastics at levels far exceeding the safety limits established for consumer products. Due to the wide development of novel materials [110,111,112,113,114] (e.g., novel conducting polymers for hydrogen storage), the risks of traces of impurities is highly underestimated. Addressing topics of current state-of-the art aspects in hydrogen storage and green, biobased polymer transition technologies, this paper goes beyond the state-of-the-art aspects in the field proposing novel standards and novel insights.

To address this oversight, it is proposed that the risks associated with environmental plastics be evaluated by comparing empirically derived mobilized metal concentrations with established migration safety limits for hazardous metals in consumer products. By adopting this approach, researchers and policymakers can better understand the potential threats to wildlife and ecosystems posed by hazardous metal contamination in plastics. Moreover, it underscores the importance of comprehensive strategies to mitigate the environmental impact of plastics and to ensure the safety of both human health and the environment.

Transitioning towards sustainability in polymer science necessitates a shift from solely relying on qualitative assessments to embracing a comprehensive array of metrics. Sustainable polymer production is a critical area of research and development, especially given pressing environmental concerns and our reliance on finite resources. The utilization of renewable resources in polymer production is on the rise.

Biopolymers extracted from natural sources are materials of the future, and those include waste or raw (i) polysaccharides: cellulose, chitin/chitosan, starch, and alginate; (ii) proteins: gelatin, silk fibroin, keratin, and soy protein; (iii) polyhydroxyalkanoates produced from the microbial fermentation of renewable feedstocks; and (iv) polylactide derived from plant-based sugars. In addition, current state-of-the-art solutions in polymer engineering enabled the synthesizing of traditional polymers (polyglycolide, poly (butylene succinate), polyolefins, poly (ethylene terephthalate), poly (ethylene furanoate), polyamides, polyurethanes, and polycarbonates) from different renewable sources. Beyond state-of-the-art investigations that cover the application of novel enzymes and their cocktails in the pretreatment or pre-processing of waste or novel polymers, researchers should strongly enhance the development of this technology in the near future.

Notably, monomers like carbon dioxide, terpenes, vegetable oils, and carbohydrates serve as feedstocks for manufacturing a diverse range of sustainable materials and products. These include elastomers, plastics, hydrogels, flexible electronics, resins, engineering polymers, and composites. Efficient catalysis plays a crucial role in monomer production, facilitating selective polymerizations, as well as enabling the recycling or upcycling of waste materials. Such sustainable polymers present opportunities for applications in both high-value sectors and basic applications such as packaging [115,116,117,118,119,120,121,122,123,124].

Encouragingly, there has been a notable rise in the adoption of such metrics and tools within the field. These encompass various items, ranging from relatively simplistic metrics like the E factor or the Toxicity Estimation Software Tool (T.E.S.T., US, EPA), which offer limited insights but are easily incorporated into existing workflows, to more robust methodologies like life cycle assessment (LCA). LCA, in particular, stands out for its thoroughness, although it requires more effort to be implemented effectively.

The decision on which protocol to use, therefore, will depend on the properties of the sample, the amount of the sample available for analysis, the costs, and all other vital information that will dictate the steps in the chemical analysis, particularly the ability for achieving very low limits of detection.

## 6. Conclusions

In conclusion, the challenges associated with transitioning to green practices in polymer production are multifaceted but hold significant promise for advancing sustainability in various industries. The development and application of innovative technologies, such as zero-energy innovations and hydrogen storage, offers avenues for reducing environmental impacts and promoting renewable energy sources. By leveraging renewable resources and efficient catalysis, there is potential to produce polymers with improved properties and reduced carbon footprints. However, addressing the challenges of scalability, cost-effectiveness, and compatibility with existing infrastructures remains critical for widespread adoption. Collaboration among researchers, industry stakeholders, and policymakers will be essential in overcoming these challenges and realizing the full potential of green transitions in polymer production. Through collective efforts and continued innovation, we can pave the way for a more sustainable future in polymer science and beyond. The goal of this paper was to pin out some topics that are often underestimated, but that may have a pivotal role in the future. Zero-waste energy represents a paradigm shift towards a more sustainable, circular economy where waste is viewed as a valuable resource rather than a burden. By embracing the principles of waste reduction, reuse, recycling, and energy recovery, societies can mitigate environmental pollution, reduce their reliance on finite resources, and mitigate greenhouse gas emissions. However, realizing the full potential of zero-waste energy requires concerted efforts from policymakers, industry stakeholders, and civil society to overcome barriers and capitalize on opportunities for innovation and collaboration. Ultimately, the journey towards a zero-waste energy future is not just a technological imperative but a moral and ethical imperative to safeguard the planet for future generations.

Sustainable polymers offer a viable and environmentally friendly solution for hydrogen storage, addressing the critical need for efficient and safe energy carriers in the transition towards a sustainable future. Despite the existing challenges, ongoing research and technological advancements are driving progress towards the practical implementation of polymer-based hydrogen storage systems. By leveraging the tunable properties, versatility, and sustainability of polymers, researchers can contribute to the development of innovative storage solutions that enable the adoption of hydrogen as a powerful and potential clean energy source.

## Figures and Tables

**Table 1:** Average toxic elements in polymeric dyes.

Element	Anionic	Cationic	Substansive
Arsenic	Less than one ppm	Less than one ppm	Less than one ppm
Cadmium	Less than one ppm	Less than one ppm	Less than one ppm
Chromium	Less than 10 ppm	Less than three ppm	Less than three ppm
Lead	Less than 40 ppm	Less than seven ppm	Less than 30 ppm
Mercury	Less than one ppm	Less than one ppm	Less than one ppm
Nickel	Less than 15 ppm	Less than 35 ppm	Less than 10 ppm

**Table 2:** Interactions and accumulations of toxic metals in particular parts of body [23] *.

System	Arsenic	Cadmium	Cobalt	Mercury	Lead
Circular		+	+		+
Digestive	+	+	+	+	+
Hormonal	+		+		+
Immune			+	+	+
Neural	+	+	+	+	+

* the toxic metals that accumulate and interact with only one system in a human body are chromium (the digestive system) and nickel (the immune system), which are therefore not presented in Table 2.

**Table 3:** List of health-concerning metals according to the TOX Proof Standard.

Element	Element Symbol	Heavy Metal μg/mL
Antimony	Sb	0.2
Arsenic	As	0.2
Cadmium	Cd	0.1
Chromium	Cr	0.0
Cobal	Co	1.0
Coper	Cu	20.0
Lead	Pb	0.8
Mercury	Hg	0.02
Nickel	Ni	1.0
Zinc	Zn	20.0

**Table 4:** Öko–Tex Standard list of parameters and their prescribed limits for extracted compounds.

Important Parameters of Extracts from Polymers	Limit	Important Parameters of Extracts from Polymers	Limit
pH	4.8–7.5	Halogenated carriers	Bellow limit of detection
Saliva (baby items)	Resistant	Carcinogenic dyes	Bellow limit of detection
Perspiration	3–4	Sensitizing dyes	Bellow limit of detection
Washing	3–4	Pesticides (/ppm)	Less then 1 ppm
Water, severe	3	Total content	
Crocking (dry/wet)	4/2–3	Aldrin	Less than 0.2 ppm
Heavy metals	/(ppm)	Dieldrin	Less than 0.3 ppm
Antimony (Sb)	30	2,4–D	Less than 0.1 ppm
Arsenic (As)	0.2–1.0	2,4,5–T	Less than 0.05 ppm
Cadmium (Cd)	0.1	DDT	Less than 1 ppm
Chromium six (Cr(VI))	Not detectable	HCH	Less than 0.5 ppm
Chromium three (Cr(III))	1.0–2.0	Heptachlor	Less than 0.5 ppm
Cobalt (Co)	1.0–4.0	(epoxide)	
Copper (Cu)	25.0–50.0	Lindane	Less than 1 ppm
Lead (Pb)	0.2–1.0	Toxaphen	Less than 0.5 ppm
Mercury (Hg)	0.02	Emission of volatile	Very limited for
Nickel (Ni)	1.0–4.0	substances	indoor polymers
Formaldehyde	20/75/300	Strange odor	Limited

**Table 5:** Metals in plastics with prescribed limits according to the following directives: the Toy Safety Directive (for migration limits in hydrochloric acid), and in the directive for Packaging and Packaging Waste, the combined limit for Cd + Cr + Hg + Pb metals is 100 mg kg^−1^ [100,101,102,103,104,105,106,107,108,109].

Limits Expressed in mg kg^−1^	TS
As	Less than 50
Cd	Less than 20
Cr(IV)	Less than 0.3
Co	Less than 150
Hg	Less than 160
Pb	Less than 25

## Data Availability

Data are contained within the article.
